# Evolving Roles for Patients as Partners in a National Kidney Health Research Network: A Qualitative Study

**DOI:** 10.1111/hex.70710

**Published:** 2026-06-04

**Authors:** Mark Melika‐Abusefien, Nicolas Fernandez, Keila Turino Miranda, Jocelyn M. Jones, Melanie D. Talson, Letitia Pokiak, Selina Allu, Julie Wysocki, Matthew T. James, Meghan J. Elliott

**Affiliations:** ^1^ Department of Medicine University of Calgary Calgary Alberta Canada; ^2^ Department of Family Medicine and Emergency Medicine Université de Montréal Montréal Québec Canada; ^3^ Department of Kinesiology and Physical Education McGill University Montreal Québec Canada; ^4^ Can‐SOLVE CKD Network Vancouver British Columbia Canada; ^5^ The Kidney Foundation of Canada Montreal Québec Canada; ^6^ KRESCENT Program Montreal Québec Canada; ^7^ Department of Community Health Sciences University of Calgary Calgary Alberta Canada

**Keywords:** patient engagement, patient‐oriented research, qualitative study, roles

## Abstract

**Introduction:**

Patient partnerships are central to all activities of the patient‐oriented kidney health research network, Canadians Seeking Solutions and Innovations to Overcome Chronic Kidney Disease (Can‐SOLVE CKD). The aim of this study was to characterise how patient partner roles in the network and the structures that influence their engagement have evolved over time.

**Methods:**

We conducted a secondary analysis of two qualitative datasets involving focus groups and interviews exploring perspectives on patient engagement within the network (Study A, *N* = 48) and a series of workshops defining network strengths and priorities related to inclusivity, diversity, equity and accessibility pillars (Study B, *N* = 49). We analysed all data from the primary studies, including deidentified transcripts (Study A) and virtual ‘sticky notes' (Study B), using inductive thematic analysis, which involved coding data in duplicate and developing themes that captured shifts in perspectives on patient partner roles.

**Results:**

Data from a total of 97 participants (40 patient partners, 43 researchers and/or clinicians and 14 network operational staff) were included in this secondary qualitative analysis. We identified three main themes: (1) Shift from traditional to emerging roles—initial roles defined by engagement frameworks were superseded by novel roles involving advocacy, outreach and mentorship; (2) Distinguishing role delegation from self‐determination—roles became increasingly active and shaped by patient partner‐identified gaps or interests; (3) Strengthening supports for meaningful patient contribution—structures for meaningful engagement grew more formalised and emphasised integration of diverse perspectives.

**Conclusions:**

The shift in roles and supports for patient partners within the Can‐SOLVE CKD Network reflects a broader movement toward more collaborative, influential and equitable research partnerships. Our findings have implications for large‐scale research teams and networks seeking to foster and sustain authentic patient engagement over time.

**Patient and Public Contribution:**

This study was undertaken in collaboration with partners with experience of kidney disease (N.F.), Indigenous advocacy related to kidney health (L.P.) and kidney policy and program development (J.W.), as well as Can‐SOLVE CKD network staff and committee members (K.T.M., J.M.J., M.D.T., S.A.). N.F. conceived and co‐led data collection and analysis in the original qualitative study. L.P. and J.W. were working group members for the IDEA project. The patient partners and network members contributed meaningful insights that shaped our approach, interpretation and presentation of findings from this secondary qualitative analysis.

## 1 Background

Patient engagement has become a growing area of interest among healthcare researchers to improve the relevance, reach and impact of health research [1]. It reflects a shift from a historical view of patients as subjects or participants to one of active, reciprocal partnership throughout the research cycle [1, 2]. Several frameworks offer guidance and practical strategies for meaningfully engaging with patients and the public in health research [3, 4, 5], though the type, timing and extent of engagement can vary widely depending on the goals and the preferences of those involved [6]. Among reported challenges to patient engagement are researcher uncertainty on how to integrate patients' voices in an effective, meaningful and nontokenistic way and low public awareness of what engagement actually entails [7, 8, 9]. As patient engagement is an inherently collaborative endeavour, the roles that patients assume and how they are supported in those roles are critical considerations from the outset of the research partnership [10].

The Canadians Seeking Solutions and Innovations to Overcome Chronic Kidney Disease (Can‐SOLVE CKD) Network is one of five pan‐Canadian chronic disease networks supported by the Canadian Institutes of Health Research (CIHR) through its Strategy for Patient‐Oriented Research (SPOR) [11]. By fostering partnerships between people with experience of kidney disease (i.e., patients, their care partners and living kidney donors), researchers and healthcare professionals across phases of research, the network aims to improve uptake of research evidence into practice and health outcomes [12]. To date, Can‐SOLVE CKD has funded and facilitated 27 distinct patient‐oriented research projects across Canada spanning kidney health populations and research pillars (i.e., basic science, translational and clinical research) [13]. While the network's first funding phase (2016–2021) focused on generating new knowledge and establishing a robust platform for patient engagement [14, 15, 16], its second phase (2022–2026) has concentrated on mobilising research knowledge into kidney care practice and policy [17, 18]. Yet, despite the centrality of patients to the network's mandate, their specific contributions to network initiatives and the extent to which these may have shifted alongside evolving network priorities have not been assessed.

A lack of formalised guidance initially on how to effectively engage in diverse, multi‐disciplinary partnerships meant that Can‐SOLVE CKD teams largely devised their own processes for determining roles and responsibilities [19]. Although existing scoping reviews provide preliminary descriptions of patient partner activities in research [20, 21, 22], limited primary research has assessed how patient partners function within research teams, how their roles are operationalised and how the scope of their engagement may change over time. With reference to two primary studies conducted at different timepoints within the network examining experiences of patient engagement and application of equity and inclusivity principles, respectively, we undertook this secondary qualitative analysis to address a distinct research question: What have been the roles of patient partners and the structures influencing their engagement over time in a variety of patient‐oriented, kidney health research initiatives within the Can‐SOLVE CKD Network?

## 2 Methods

### 2.1 Study Design and Setting

We conducted a secondary analysis of two qualitative datasets collected by the authors from distinct studies undertaken within the Can‐SOLVE CKD Network. The methodological orientation of this secondary analysis was qualitative description, which is a pragmatic approach suited to questions relevant to practice and policy and has been applied in secondary qualitative analysis in other settings [23, 24, 25, 26]. The first dataset (Study A) was derived from an exploratory qualitative study aiming to explore patient partners' and researchers' experiences of patient engagement and involving focus groups and interviews with 48 network members conducted in the first network phase (2019–2020) [19]. The second dataset (Study B) was collected over a series of seven strategic planning workshops held during the second network phase (2023–2024) to assess the views of 49 patient partners, researchers and network staff on the application of pillars of inclusivity, diversity, equity and accessibility (IDEA) [27]. Ethical approval was obtained through the Conjoint Health Research Ethics Board at the University of Calgary for the primary qualitative study (REB18‐0131) and secondary analysis (REB24‐0995); ethical approval was waived for the workshop sessions as they were conducted for internal operational purposes and no identifiable data were collected.

### 2.2 Participants

Study A included adult (≥ 18 years) patient partners and researchers with roles on ≥ 1 research project and/or committee within the network. The term ‘patient partner' referred to an individual with personal experience living with CKD, their caregiver, or a living kidney donor. Eligible researchers included principal investigators (many of whom were also healthcare professionals), research support staff and/or research trainees. Participants were sampled purposively using a maximum variation approach to capture perspectives across a range of demographic characteristics (e.g., sex/gender, geographic location), network roles and CKD‐related experiences. Study B included adult (≥ 18 years) patient partners, researchers, clinicians and staff serving in any capacity on project teams, committees or core support services within the network. Recruitment for both primary studies took place through email invitation sent to eligible participants through network communication channels, as well as snowball sampling and verbal invitation through network events. Study A included 24 patient partners and 24 researchers and Study B included 19 patient partners, 17 researchers/clinicians and 13 network staff.

### 2.3 Data Collection

Study A collected data through in‐person focus groups (2 for patient partners; 2 for researchers) and 28 individual, semi‐structured interviews (12 patient partners; 16 researchers) conducted by telephone or virtually using Zoom™. Question guides explored experiences, expectations and perceived challenges related to patient engagement in network activities. Two investigators led all focus groups and interviews (M.J.E., N.F.), which lasted 90 min and 40–60 min, respectively. Study B involved a series of virtual workshops held on Zoom™ with a total of 49 participants from 3 distinct groups: one with operations staff (14 participants), 2 with patient partners (16 participants) and 3 for clinicians and researchers (19 participants). Each workshop lasted 1.5–2 h and was led by an experienced facilitator (K.T.M.) who referred to discussion prompts on strengths and opportunities for integrating IDEA principles into network activities. While question guides from both studies did not explicitly address roles, this concept emerged frequently and without prompting during discussions. Interviews and focus groups from Study A were audio‐recorded and transcribed verbatim, whereas data from Study B were comprised of de‐identified ‘sticky notes' to document ideas by participants in real‐time using the Mural® visual collaboration platform (https://mural.co). Participants in both studies completed a demographic questionnaire to summarise the sample. All transcripts and sticky note data were uploaded to NVivo 14 (Lumivero, 2023) to facilitate data organisation, retrieval and coding.

### 2.4 Data Analysis

In both primary studies, thematic analysis was used as the main method to inductively code, synthesise and present cohesive patterns in the data to address their research objectives [28]. In this secondary analysis, a similar thematic analysis approach was used to analyze pooled data from the two studies and address the secondary research question. All data from the primary studies were included in this analysis, with each interview or focus group/workshop representing a distinct unit of analysis. Two team members (M.M., M.J.E.) first read all de‐identified transcripts and sticky notes in entirety to develop initial impressions of the dataset in relation to the new research question. They then reviewed coded data extracts from Study A within their originally assigned coding labels, which encompassed concepts related to patient partner activities, team dynamics, network resources, needs/gaps and perceived value. Coded extracts relevant to this study's objectives were retained and organised with reference to fundamental concepts of role theory, including function, context, social position and expectations [25]. Sticky note data from Study B were then coded using the same coding scheme from Study A to capture patterns in the data consistently across the pooled dataset. All coded extracts and relationships between concepts were examined to develop initial themes related to roles based on meaningful clusters and with a focus on changes over time. The two analysts met regularly to refine themes, which were discussed among the broader research team to provide complementary or reinforcing perspectives, ensure relevance of findings and enhance our thematic reporting.

### 2.5 Rigour and Reflexivity

We took steps to ensure rigour in secondary qualitative data analysis, including ensuring fit of the research question with the primary datasets, re‐coding primary data sources to address the new research question, using researcher and data triangulation, and maintaining an audit trail of all steps [29]. Our multidisciplinary research team had varied experiences and relationships to Can‐SOLVE CKD. The lead analyst (M.M.), a medical student with limited exposure to patient‐oriented research and qualitative methods, brought openness to the data with close supervision by a clinician scientist with both content and methodological expertise, as well as collaborations within the network (M.J.E.). The team included patient partners with experience of kidney disease (N.F.) and Indigenous advocacy related to kidney health (L.P.), as well as network operational staff, all of whom were involved in the primary studies and provided input into thematic development in this secondary analysis. Reflexive discussions, iterative analysis and engagement with patient partners supported critical examination of assumptions and ensured findings remained grounded in participants' accounts.

## 3 Results

Twenty‐eight interviews, four focus groups and six workshops involving a total of ninety‐seven participants were included in this secondary analysis. Of these participants, 40 were patient partners, 43 were researchers and/or clinicians and 14 were network operational staff. Two‐thirds identified as female/woman (*n* = 64), and most were under 65 years of age (*n* = 79) (Tables 1 and 2). In Study A, over half were involved in ≥ 2 network research projects and/or committees at the time of participation (*n* = 26), and most were from Western Canada (*n* = 30) or Ontario (*n* = 16). In Study B, less than half of participants self‐identified as a nonwhite race/ethnicity (*n* = 14) and/or as an Indigenous person of North America (*n* = 9).

**Table 1:** Demographics of participants from Study A (*n* = 48).

Characteristics	Number
Role in network	
Patient partner	24
Researcher	24
Sex	
Female	31
Male	16
Prefer not to answer	1
Age (years)	
< 40	9
40–64	35
≥ 65	4
Location of residence	
Western Canada	30
Ontario	16
Quebec	0
Eastern Canada	1
Territories	0
Prefer not to answer	1
Duration in Can‐SOLVE CKD Network (years)	
≤ 1	5
2–3	20
> 3	23
Number of projects and/or committees	
1	21
2–3	18
> 3	8

**Table 2:** Characteristics of participants from Study B (*n* = 49)*.

Characteristics	Number
Role in network	
Patient partner	16
Researcher and/or clinician	19
Operational staff	14
Gender	
Woman	33
Male	8
Prefer not to answer	1
Gender identity	
Cisgender	40
Two‐Spirit	1
Prefer not to answer	1
Age (years)	
18–24	3
25–34	5
35–44	8
45–54	13
55–64	6
≥ 65	7
Identifies as an Indigenous person of North America	
Yes	9
No	31
Prefer not to answer	2
Race/ethnicity	
White	23
South Asian/East Indian	3
Southeast Asian	2
Chinese, Filipino	1
Black	1
Other	7
Prefer not to answer	5
Sexual orientation	
Heterosexual	34
Gay	1
Queer	1
Bisexual	1
Another sexual orientation	1
Prefer not to answer	4
Identifies as a person with disability	
Yes, ongoing medical condition/chronic illness	8
Yes, physical disability or impairment	7
No	26
Prefer not to answer	1

* Demographic data available for 42 of 49 participants.

**Figure 1:**
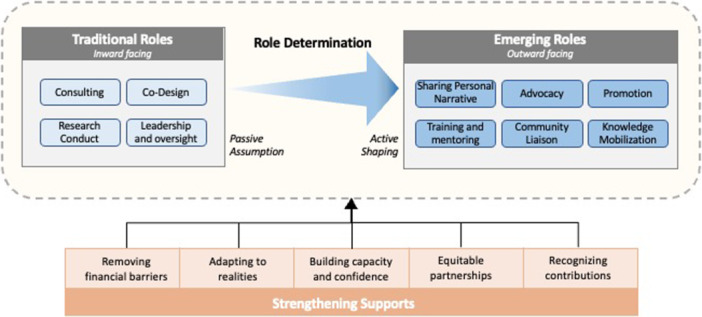
Graphical depiction of relationships between themes. A shift was noted from traditional, inward‐facing patient partner roles to emerging, outward‐facing roles over time. Whereas earlier roles were more passively assumed, later roles were identified and shaped by patient partners themselves and strengthened through increasingly formalised network supports.

We characterised three overarching themes with subthemes capturing perceived roles and their evolution over time within Can‐SOLVE CKD: (1) Shift from traditional to emerging roles; (2) Distinguishing role delegation from self‐determination; (3) Strengthening supports for meaningful patient contribution. Supporting quotes (with study identifiers) for themes and their key concepts are included below and in Table 3. Relationships between themes are depicted schematically in Figure 1.

**Table 3:** Additional participant quotes supporting each theme and subtheme.

Themes and sub‐themes	Quote
Theme 1: Shift from traditional to emerging roles	
Inward‐facing traditional roles—*Established early to advance research objectives with reference to existing patient engagement frameworks*	
Consulting—*advise on research direction and activities*	‘Maybe giving them stuff to read and review isn't always the best use of their time either. Because a lot of times that's already done once they've gotten past a certain point in the research’. (Study A—Patient 12)
Co‐design—*create protocols, processes and materials*	‘Patients have created some documents, like, cartoons that will go to dialysis units to answer questions about [symptoms]… I am not a person to develop a really amazing infographic that the patient will want to use and is meaningful to the patient. I'm not good at that. Let's get the real people in the room who really need to be focused on that. That's what I want to see in the next five years, is that we stop trying to do things for the patients and the patients are doing them’. (Study A—Researcher 10)
Research conduct—*assist with recruitment, data collection, analysis and/or interpretation*	‘We undertook the analysis collaboratively, and so for me, that makes a big difference’. (Study A—Patient 4) ‘So, I envisioned that it would happen, you know, [patient partners] as collaborators and working together. I didn't envision that they would be… reviewing transcripts and those sorts of things. So, that's been very helpful and fantastic, actually’. (Study A—Researcher 1)
Governance—*chair committees or co‐lead projects*	‘The patient leads really need to be, like, a whole part of the project management. I know that's not our job, that's not our day job… I guess the thing is it's all evolving. We're building towards that. I don't think we're there yet, but I think it's coming’. (Study A—Patient 5) ‘All aspects of medical care and research—Can‐SOLVE has done a great job at putting pts at the forefront’. (Study B—Clinician/Researcher)
**Outward‐facing emerging roles**—*Created later into partnership to advance community linkages, patient supports and research uptake*	
Sharing personal narrative—*provide insight into lived experience of CKD*	[Interviewer: ‘What do you think patients can contribute to research teams?‘] Response: ‘Our personal stories. Our first‐hand stories… I think that's something that, this is my own opinion, it took a while for researchers to realize that we had something to offer outside of being a patient’. (Study A—Patient 12) ‘Patient voice and lived experience in all phases of the research – ensures the research we do meets the needs of clinical populations adds richness and direction’. (Study B—Clinician/Researcher)
Training and mentoring—*develop training resources, support other patient partners*	‘I was a first‐generation transplant ambassador [for Can‐SOLVE CKD project], so in turn I train transplant ambassadors now’. (Study A—Patient 4)
Advocacy—*amplify patient and community voices in the health system*	‘I just think that that's a piece that's missing, where they can actually talk to an everyday person who's not a medical person and feel like, ‘Well, you're a real person, I can actually ask you questions'’. (Study A—Patient 2) ‘Promote indigenous voices to promote advocacy for CKD prevention’. (Study B—Patient)
Community liaison—*facilitate outreach and linkages with patient communities*	‘We want to establish relationships with these [Indigenous] communities. We want some of them to be the communities that we're connected to and doing those things, so we want to really establish those’. (Study A—Patient Focus Group 1) ‘Get information where non‐urban communities lie – Where are they? What's the best way to reach them?’ (Study B—Patient)
Promotion—*spread awareness of network activities among the public*	‘She [patient partner] engaged with other patients that she was aware of and knew of within [Province] that are families or caregivers that have CKD. And then, I had other patients or patient family members approach me to see how they… can get involved more broadly in research or giving back to kidney communities. And so, I made that opportunity available to them’. (Study A—Researcher 5)
Knowledge mobilisation—*enhance research dissemination and uptake*	‘A lot of the science we come up with is not always straightforward. There are very few studies now that come with these blockbuster results… and I think it's important then to be able to put it in language that is clear, that the information is written so that people can understand it and then see how they can integrate this into their care, and how they can also share any results’. (Study A—Researcher 14)

Theme 2: Role delegation versus self‐determination	
Passive role assumption—*Roles delegated to and/or simply taken on by patient partner, often based on project or researcher need*	
Assignment of roles—*predetermined by researcher, selection from ‘menu’*	‘Initially I would just ask, ‘What would you like to do?', and they [patient partners] came back to me with silence because they didn't know what the options were’. (Study A—Researcher Focus Group 1) ‘You're just kind of put in there and don't get any training, so I had no idea… what am I doing here?’ (Study A—Patient Focus Group 1)
Engagement constrained—*limited role variety and creativity*	‘Categorization of a patient partner is limiting (perspectives)’. (Study B—Patient)
Stepping up—*filling a gap, fluid and opportunistic*	‘Like, when I'm talking to the research team, if there's anything that need to be done, you know, I'm willing to do it. I'll do it right now and support them in any way’. (Study A—Patient 5) ‘I'd say those are the main ways that patients roles are determined, primarily by the research team, but also some opportunistic opportunities for involvement’. (Study A—Researcher 12)
**Active role shaping**—*Roles driven by patient partner interest or expertise, with emphasis on diversity, reach and impact*	
Defining own roles—patient partner carves out a new role for themself	‘I've been actively trying to get them to adopt the kidney equation or the risk equation in our own health records. So, I guess I'm creating my own role there’. (Study A—Patient 5)
Alignment with expertise—leveraging an existing skillset, interest or experience	‘He's a computer programming person for digital health, and we talked about one of the limitations in using clinical decision‐making in medicine is that the tools are not directly integrated into EMRs. So, he independently, on his own, started having conversations with people out there and he's trying to facilitate the integration of our tools outside of the trial in [Province]. He made the connections because he has the technical know‐how’. (Study A—Researcher 8) ‘Originally, as a patient partner I was just asked what projects jumped out at me and were interesting, but then also asking what some of my own skills were that I might be able to offer to the group, and then also what skills I was looking to build upon’. (Study A—Patient 9)
Expanding diversity and reach—ensuring inclusion of underrepresented voices	‘Perspectives from other groups—cultural/ethnic populations; gender‐diverse; disability’ (Study B—Researcher/Clinician). ‘We are only hearing from who is at the table and not those that are not. Need to reach into these underrepresented communities’. (Study B—Operational Staff)

Theme 3: Strengthening supports for meaningful patient contribution	
Removing financial barriers—*Provision of fair compensation for patient engagement in research*	
Reimbursement protocols—*harmonising practices*	‘I'm quite comfortable with the level of engagement with our patients and so on, and I think I like the idea of paying patients some nominal reimbursement. I think they should be part of the team’. (Study A—R5)
Equitability and transparency—*enhancing inclusion of diverse communities*	‘Policy for travel companions to be compensated when accompanying patient partners/knowledge keepers with mobility/accessibility needs’. (Study B—Operational Staff)
**Adapting to patients' realities**—*Accommodating other commitments and health needs*	
Competing priorities and burden—*not overtasking patient partners*	‘One of our patient leads, she is on the main patient engagement committee and the national committee, so she does that. But then, when it comes to other asks, we don't want to overwhelm her. She has a full‐time time job, so we will engage with other co‐leads’. (Study A—Researcher 14). ‘Heavy workload—hard to add onto that’. (Study B—Patient)
Unexpected health decline—*accommodating absences and offering support*	‘Safe space for chronically ill and disabled team members to ask for accommodations and no need to pretend you aren't sick/disabled’. (Study B—Operational Staff)
**Building capacity and confidence** – *Enhancing research readiness among patient partners*	
Tailored training—enhance skills in research and patient engagement	‘I think that part of the evolution is making more training more available for patients’. (Study A—Patient 12)
Peer mentorship—patient‐to‐patient supports within network	‘Connect new patient partners with existing patient partners’. (Study B—Patient) ‘Support system allows collaborative resolution’. (Study B—Patient)
Patient partner recruitment—expand pool of engaged patients	‘I think sometimes it might be the same patients for all the projects that keep volunteering because they're very active, but you might be missing some others. So, I think that will be a continued issue that we'll have to try to solve to figure out how best to recruit them and recruit different kinds of patients’. (Study A—Researcher 6) ‘Finding out about Can‐SOLVE CKD—not apparent it's an organization you can join’. (Study B—Patient)
**Collaborative and equitable partnerships**—*Respectful interactions and a safe, inclusive research environment*	
Nonhierarchical structure—perceived equality and comfort contributing	‘When they [patient partners] come and attend meetings, they are very much like equal partners, in terms of everyone sitting in the room, and I think that a lot of their feedback is almost taken automatically. There's never a doubt on: “Oh, should we include this or not?” Like, it's almost automatic, just respecting the fact that they are making very valid points’. (Study A—Researcher 3) ‘Being treated fairly and encouraged to thrive and do our best work’. (Study B—Operational Staff)
Foundations of trust—authentic relationships, reciprocity and cultural safety	‘In my culture, relationships are really important… relationships are reciprocal, you give back and forth’. (Study A—Patient 12) ‘I have a very good relationship with the research manager in all of those things, and I think… they [researchers] don't only care about you from a research perspective, they care about you from a life perspective’. (Study A—Patient Focus Group 2)
**Recognising patient contributions**—*Demonstration of appreciation and value to patient partners*	
Nonfinancial acknowledgement—for example, co‐authorship, presentations, certificates, tokens of appreciation	‘One of the things was just, like, “Well, it would be nice if you kind of got a certificate of your contribution to this project to show that I contributed to this work” or something like that. We need to do those things‘. (Study A—Researcher Focus Group 1) ‘Each of these 18 projects will have paper published, and in the ideal world patient are co‐authors on these papers. I have already been a co‐author on several papers to do with [our] project. That is where we should be going, that's the way research should be thinking of our input’. (Study A—Patient Focus Group 1)
Conveying value—expressions of gratitude, sense of meaning and impact	‘But that recognition piece, like, I think about that one patient partner that phones me, right, and she just wants to hear, for me to directly answer her questions, and then she feels recognised with just that conversation’. (Study A—Researcher Focus Group 1) ‘Several of our patient partners say, “Actually, in some of the studies, what we like is that we get you all to ourselves outside of the normal busy clinic stuff, and we can have an entirely different experience, which makes me feel like it's really special. Like there's something that's really been added to our care by doing it”.' (Study A—Researcher 9) ‘Equitable recognition of individual team members' efforts and achievements [future direction]’ (Study B—Operational Staff)

### 3.1 Shift From Traditional to Emerging Roles

#### 3.1.1 Inward‐Facing Traditional Roles

Participants described more traditional roles for patient partners early into the network's existence and upon their initial integration into research activities. These roles were considered inward‐facing as their main goal was to drive the network's research mandate forward and ensure, ‘Does it resonate with [patients]?’ (Study A—R1). Many discussed patient partners advising on operational aspects of research (e.g., enhancing accessibility of patient‐facing materials) but noted that ‘giving them stuff to read and review isn't always the best use of their time’ (Study A—P12). Participants also highlighted patient roles in co‐designing research protocols and, in some instances, engaging in research conduct with support, such as participant recruitment, data collection and analysis/interpretation of results. While participants also discussed patient involvement in research oversight, including project and committee co‐leadership, several expressed initial uncertainty about their expected contribution and desire for greater influence. For example:

I hope within 5 years that patient partners will be active members of grants, so they could sit as a co‐lead or play a much larger official roles on a research project.

(Study A—R3)

#### 3.1.2 Outward‐Facing Emerging Roles

Participants in both studies indicated several novel roles for patient partners that typically arose later and were often outward‐facing, meaning they extended beyond the insular activities of the network to build awareness, linkages and capacity among the broader CKD community. Examples ranged from patient involvement in training and mentoring other patient partners in research, to serving as intermediaries between the network and patient and/or clinical communities to promote greater awareness and uptake of the research. These roles provided ‘more opportunities for patients to talk to patients’ (Study B—Patient) and helped researchers ‘realize that [patients] have something to offer outside of being a patient‘ (Study A—P12). With respect to engagement of Indigenous communities, participants discussed the crucial roles for members of those communities in ‘not just acting as a liaison, but also building those relationships’ (Study A—FG‐P1) and a solid foundation of trust. Common across these emerging roles was an emphasis on strengthening connections with individuals and groups affected by CKD through ‘champions who support and [provide] outreach’ (Study B—Patient) and planning for sustainability of the network and its innovations.

### 3.2 Distinguishing Role Delegation From Self‐Determination

#### 3.2.1 Passive Role Assumption

Participants described the integration of patient partners into early network activities as mostly passive, whereby they were assigned roles ‘determined primarily by the research team’ (Study A—R12) based on project need and with limited scope. Participants also relayed instances of patient partners stepping in to fill gaps or seize opportunities that arose, with one describing the ideal patient partner as someone ‘willing to participate in a variety of ways’ (Study A—P4). However, this approach was generally considered ‘limiting’ (Study B—Patient) and prompted some patients to question the authenticity of their involvement when it was reduced to narrowly defined, supporting tasks. Some participants identified how a more passive approach to defining roles may be preferred or driven by a lack clarity about how to contribute. As one researcher said:

Sometimes a patient just wants to sit there and soak it up, and maybe talk a little but not really be actively involved.

(Study A—FG‐R2)

#### 3.2.2 Active Role Shaping

As network activities progressed, participants described how patient partners became active in defining their own roles and responsibilities within the network. Often stemming from a desire for ‘real, authentic partnership and having a voice’ (Study A—P10), participants in both studies conveyed a progression from simply selecting roles from a menu of options to creating new roles for themselves. This shift challenged assumptions that some researchers held about how patients could contribute to research, with one patient partner noting, ‘Well, you won't know that until you actually ask us’ (Study A—P3). While many of the patient‐driven roles intended to make use of their unique skills, experiences or interests, they also suggested that role creation is as much about equity and representation as it is about personal agency. Several comments during IDEA workshops called for greater diversity across roles and the need for ‘identification of where voices are missing and addressing these gaps’ (Study B—Patient).

### 3.3 Strengthening Supports for Meaningful Patient Contribution

#### 3.3.1 Removing Financial Barriers

Participants from Study A discussed the financial supports available to patient partners since the network's inception, guided by its funding terms and protocols. These typically involved cash or gift cards as reimbursement for expenses incurred (e.g., travel) or compensation for time spent on network‐related tasks. Patients noted how these supports helped offset the opportunity costs of ‘taking time off without pay to attend meetings’ (Study A—P6), though offers of payment were not always accepted. Alongside network efforts to promote greater inclusivity, participants in both studies remarked on how adaptations to remuneration procedures should better consider the needs of socially or medically disadvantaged groups. For example, one IDEA workshop participant suggested a ‘policy for travel companions to be compensated’ (Study B—Operational Staff) for patient partners with accessibility needs.

#### 3.3.2 Adapting to Patients' Realities

Given the challenges patient partners face in living with CKD, participants emphasised how their medical circumstances and life priorities must be accommodated when establishing roles in network activities. One participant noted that patient involvement is ‘time intensive… patients have other commitments’ (Study B—Researcher/Clinician) and that competing priorities may not become apparent until after the work begins. Commonly discussed accommodations included flexible meeting scheduling (e.g., after‐hours, virtual/telephone), avoiding overtasking patient partners and responding with compassion when they cannot fulfil their responsibilities due to unanticipated events, such as a decline in health. In addition to ‘including more patient partners to share the workload’ (Study B—Researcher/Clinician), some described relying on patients to indicate when and what accommodations they might require:

I'm very aware of not putting too much of a burden on people, but I don't want to dictate that… that's up to them to say.

(Study A—R10)

#### 3.3.3 Building Capacity and Confidence

Participants described how initial concerns about patient partners' ‘limited understanding’ (Study A—R5) of the kidney health research landscape and researchers' lack of know‐how in patient engagement prompted the network to develop a variety of training resources on topics such as patient‐oriented research, knowledge translation, storytelling and cultural safety. They indicated these resources helped to ‘remove barriers of participation through education’ (Study B—Patient) and empower patients to take on more active and informed roles. Expanding efforts to recruit new patient partners and formalising mechanisms for peer mentorship were also suggested to enhance patient partner capacity, confidence and sense of belonging across network activities. As one patient said,

Sometimes it's hard, and I just wish I had… another patient partner I could connect with that was in my situation.

(Study A—P7)

#### 3.3.4 Collaborative and Equitable Partnerships

When embarking upon patient‐oriented research, patients described expecting a ‘top‐down’ structure but ‘found that not to be the case’ (Study A—P4). Researchers and network staff attributed this to steps taken to ensure a ‘non‐hierarchical [and] supportive environment’ (Study B—Operational Staff) in which ‘people are able to share more freely without fear of being judged’ (Study A—P11). They noted how foundations of trust and mutual respect were established early across the network and empowered patient partners to contribute in increasingly transformative roles. A commitment to reciprocity and culturally safe engagement, including respect for Indigenous ways of knowing and community‐centred care values, was identified as essential for meaningful involvement and prioritised as a direction for the network:

Promoting indigenous cultural respect and safety at the forefront of CKD advocacy and general health.

(Study B—Patient)

#### 3.3.5 Recognising Patient Contributions

Acknowledgement of patient partner contributions was described as a way of encouraging them to remain motivated and active in their roles. In addition to the remunerative supports above, participants indicated how recognition often took the form of authorship on scientific reports, involvement as presenters at meetings and tokens or expressions of appreciation. However, some expressed uncertainty about the optimal mode of recognition and pointed to the network's ongoing efforts to harmonise such processes. For example:

Equitable recognition of individual team members' efforts and achievements [future direction].

(Study B—Operational Staff)

Participants emphasised that appropriate and equitable patient partner recognition not only affirms ‘that they're making a meaningful contribution’ (Study A—R6) to CKD research and care, but that their involvement is valued by the research team.

## 4 Discussion

In this secondary qualitative analysis, we identified a shift from traditional, advisory‐based involvement of patients within a kidney health research network to more diverse, self‐directed roles that included community outreach, advocacy and mentorship, among others. This shift was supported through structural accommodations but largely driven by patient partners themselves, who took initiative through leadership roles and peer support. The evolution of patient partner roles we observed reflects a broader movement away from tokenistic involvement and toward more collaborative and influential partnerships. Our study highlights important considerations for research teams aiming to meaningfully engage patients as partners and to sustain these partnerships over time.

Our findings are consistent with a growing body of literature on how patient engagement has matured both conceptually and practically from simply carrying out prescribed tasks and absorbing research experiences to more collaborative, meaningful, and creative partnerships [30]. Results from scoping reviews indicate the roles enacted by patient partners in health research have most commonly encompassed consultation and knowledge translation activities in discrete projects, with patient partners contributing often at mid to high levels of engagement (i.e., ‘involve' and ‘collaborate') [1] yet holding leadership or principal supporting roles in < 25% of included studies [20, 21]. Similarly to other work, our findings suggest a supportive environment and increasing confidence are key drivers of patient partner ownership of their roles on the research team [31]. Given the centrality of patients to the network's mandate alongside increasing international attention to patient and public involvement in health research, Can‐SOLVE CKD has prioritised the creation of resources to support meaningful patient engagement and capacity building around patient‐oriented research methods. Examples include the KidneyPRO online training module, KidneyLink platform connecting interested patient partners with investigators, and Learning Pathway supporting culturally safe research practices (accessible through https://cansolveckd.ca) [14, 32]. In contrast to other studies conducted within single projects or at discrete time points, our findings provide insight into a breadth of roles at a network level across multiple research, governance and capacity‐building initiatives over time.

Increasingly prominent roles among patient partners in research leadership or governance can help strengthen the partnership by addressing hierarchical gaps, encouraging shared decision‐making and validating the importance of lived experience [33]. They also better position patient partners to shape the direction of research activities and advocate for the patient voice in a more influential way [34, 35]. However, roles should not be assumed merely as symbolic inclusions but based on a balance of several factors, such as patient partner interest and direction, research goals and commitment of the research team to sustain equitable, respectful partnerships [36]. In our study, trust emerged across themes as foundational to the shift toward more active patient partner roles, serving to promote both patient confidence in engaging at higher levels and researcher receptivity to shared leadership [37, 38, 39]. When not prioritised, trust between patient partners and researchers can quickly erode and result in inequitable collaboration, poor quality dialogue and ultimately unsustainable working relationships, underscoring the need for transparency and clarity around expectations for roles early in the research partnership [38].

In addition to the evolving functions of and supports for patient partners, our findings suggest an increasing attention to diversity, inclusivity and equity across network activities. Tangible opportunities to promote the integration of IDEA principles were notable in Study B given, its objective, although even in the earlier Study A, patient partners and researchers identified a strong rationale and strategies for engaging with communities historically underrepresented in research yet most likely to experience poor health outcomes, such as Indigenous communities. Even among those interested in research involvement, barriers such as lack of availability, low awareness of engagement opportunities and caregiving responsibilities may disproportionately affect underrepresented groups [40]. In one recent study, key considerations for addressing barriers to engagement among such groups, including trust, demonstration of impact and involvement from the outset, echo our study's findings and reinforce the importance of adapting the ‘how', ‘why' and ‘with whom' of engagement activities in large‐scale initiatives such as Can‐SOLVE CKD [41]. Indeed, participants from the primary studies in our analysis went beyond simply identifying a need for greater representation in kidney health research to suggesting pragmatic strategies to enhance inclusivity and diversity, such as community outreach, responsive communication, co‐leadership and culturally safe research approaches.

Our study is strengthened by its robust dataset spanning two distinct yet related studies at two time points and the involvement of patient partners and investigators from the original studies. However, we acknowledge some limitations. As this was a secondary qualitative analysis, the data were collected in the pursuit of different research questions and thus did not directly address the topic of patient partner roles. However, our analysis included all transcripts and notes from the primary studies and identified concepts relevant to perceived roles in most of them. As workshops for Study B were not recorded, the data were comprised of written ‘sticky notes' only without identifiers (beyond participant group) and lacked contextual detail, which may have influenced our coding and/or interpretation of the data. Additionally, different demographic information was collected from the two studies, and we could not discern if or how many individuals may have participated in both studies due to the de‐identified nature of the data. However, the turnover among patient partners, staff and researchers within the network and the unique focus of each study provided a large sample and breadth of perspectives captured in this analysis. Lastly, our application of a coding scheme derived from transcripts in Study A to sticky notes in Study B may have overlooked new concepts arising from IDEA workshops, although we took an additional step of reviewing all data with reference to key concepts of role theory and ensured consistency within and between coded extracts.

## 5 Conclusion

In summary, we identified a shift from traditional roles for patient partners as defined in patient engagement frameworks to active, self‐directed roles that connect Can‐SOLVE CKD research teams with communities affected by CKD and promote uptake and sustainability of health innovations. The increasing attention given by network members to IDEA across initiatives highlights important avenues for future work to address systemic and structural barriers to meaningful patient engagement and improved health outcomes.

## Author Contributions

**Mark Melika‐Abusefien:** methodology, writing – original draft, writing – review and editing, formal analysis, data curation. **Nicolas Fernandez:** investigation, methodology, writing – review and editing. **Keila Turino Miranda:** investigation, writing – review and editing, methodology. **Jocelyn M. Jones:** investigation, methodology, writing – review and editing. **Melanie D. Talson:** investigation, methodology, writing – review and editing. **Letitia Pokiak:** writing – review and editing, investigation. **Selina Allu:** investigation, methodology, writing – review and editing. **Julie Wysocki:** investigation, methodology, writing – review and editing. **Matthew T. James:** investigation, methodology, writing – review and editing. **Meghan J. Elliott:** conceptualisation, investigation, writing – original draft, methodology, visualisation, writing – review and editing, project administrations, supervision, formal analysis.

## Funding

The authors have nothing to report.

## Ethics Statement

Ethics approval for this study was obtained from the University of Calgary Conjoint Health Research Ethics Board (REB24‐0995).

## Conflicts of Interest

The authors declare no conflicts of interest.

## Data Availability

The data that support the findings of this study are available on request from the corresponding author. The data are not publicly available due to privacy or ethical restrictions. This study used individual, participant‐level data collected during interviews, focus groups and workshops. We are unable to make our dataset available due to restrictions on sharing potentially identifiable data as outlined in our Research Ethics Board certification. Inquiries related to this study's dataset can be directed to the corresponding author.
